# Emerging methylation-based approaches in microbiome engineering

**DOI:** 10.1186/s13068-024-02529-x

**Published:** 2024-07-10

**Authors:** Changhee Won, Sung Sun Yim

**Affiliations:** 1 Department of Biological Sciences, Korea Advanced Institute of Science and Technology (KAIST), Daejeon, Republic of Korea; 2 Graduate School of Engineering Biology, KAIST, Daejeon, Republic of Korea; 3 KAIST Institute for BioCentury, KAIST, Daejeon, Republic of Korea; 4 Korea Research Institute of Bioscience and Biotechnology (KRIBB), Daejeon, Republic of Korea

**Keywords:** Bacterial epigenetics, Methylome, Restriction-modification (R-M) systems, DNA methyltransferases, Microbiome engineering

## Abstract

Bacterial epigenetics, particularly through DNA methylation, exerts significant influence over various biological processes such as DNA replication, uptake, and gene regulation in bacteria. In this review, we explore recent advances in characterizing bacterial epigenomes, accompanied by emerging strategies that harness bacterial epigenetics to elucidate and engineer diverse bacterial species with precision and effectiveness. Furthermore, we delve into the potential of epigenetic modifications to steer microbial functions and influence community dynamics, offering promising opportunities for understanding and modulating microbiomes. Additionally, we investigate the extensive diversity of DNA methyltransferases and emphasize their potential utility in the context of the human microbiome. In summary, this review highlights the potential of DNA methylation as a powerful toolkit for engineering microbiomes.

## Background

The microbiome constitutes a complex ecosystem of microorganisms inhabiting specific environments, including the human body, soil, and water, where they interact significantly with diverse environmental factors. The human microbiome, consisting of trillions of microbial cells, has gained prominence as a critical determinant of human health and disease [1–3]. As a result, the demand for microbiome engineering has surged, prompting active research efforts aimed at manipulating microbial communities to achieve desired outcomes in fields, ranging from biotechnology and personalized medicine to environmental remediation and sustainable agriculture [4, 5].

To manipulate and control microbiomes, a variety of systems and synthetic biology approaches have been employed. Engineering techniques have been applied to modify probiotic or commensal bacterial species for the diagnosis and treatment of a wide range of metabolic diseases and cancers [6–9]. Horizontal gene transfer mechanisms have proven effective for directly modifying the genetic makeup of microbial populations in soil [10] and even within the mammalian gut [11]. Target specificity within Type VI secretion systems and bacteriophages has been manipulated using surface-displayed nanobodies [12] and domain-swapped receptor-binding proteins [13] to selectively deplete specific microbes within a microbial community. However, despite these advancements, precise manipulation of microbial composition and overall microbiome function remains a formidable challenge [14]. Thus, substantial innovations are required to unravel and control the microbial species profiles, spatiotemporal dynamics, and intercellular networks governing microbiomes’ emergent properties.

The emergence of evidence supporting epigenetic regulation in prokaryotes has elevated bacterial epigenetics to a pivotal dimension of microbial modulation. Among the various epigenetic mechanisms, such as nucleic acid modifications, histone-like proteins, and post-translational modifications, DNA methylation stands out as the primary regulator employed by bacteria to adapt to their environments [15, 16]. It influences both commensal and pathogenic interactions within a host [17, 18], affecting gene expression, DNA replication timing, DNA repair systems, and phase variation [19–23]. Bacterial DNA methylation typically involves adding a methyl group from S-adenosyl-L-methionine (SAM) to specific positions in target DNA bases, catalyzed by DNA methyltransferases (MTases) [24, 25]. Notably, DNA MTases often serve as integral components of restriction-modification (R-M) systems, imposing barriers to the genetic tractability of diverse microorganisms [26, 27].

The diverse molecular mechanisms responsible for epigenetic markers in bacteria could offer significant potential for modulating bacterial phenotypes in a reversible and programmable manner, without directly altering the DNA sequence itself [28]. This capability has the potential to enhance our understanding of microbiome interaction with our bodies and environments at the molecular level. Advancements in manipulating bacterial epigenetic modifications, coupled with innovative gene delivery strategies tailored to specific microbiome members could accelerate the progress of precision microbiome engineering. This review delves into the methodologies for profiling bacterial epigenomes, examining strategies used to capture and harness epigenetic modifications within bacterial genomes. Furthermore, by exploring existing applications of bacterial epigenetic modifications for microbial engineering and discussing potential applications for elucidating and modulating entire microbiomes, we consider how bacterial epigenetic systems could be leveraged in the field of microbiome.

### Methods for profiling bacterial epigenomes

Understanding bacterial epigenomes is crucial for advancing microbiome research, yet it has been less explored than eukaryotic epigenetics [28]. While studies on eukaryotes have focused on 5-methylcytosine (5mC), bacterial methylome characterization demands attention to modifications like N6-methyladenine (6mA) and N4-methylcytosine (4mC), which are more prevalent in bacteria [29]. Recent advances in high-throughput sequencing technologies have facilitated the comprehensive profiling of bacterial epigenomes, uncovering diverse genomic and epigenetic landscapes across bacterial taxa.

**Fig. 1:**
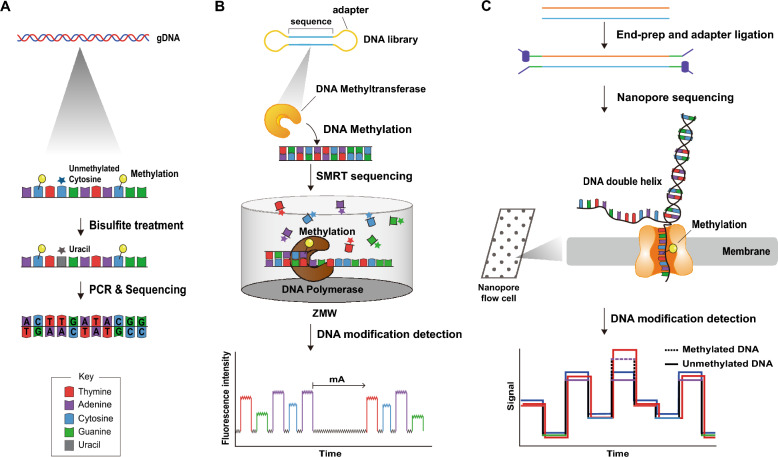
Representative sequencing technologies for mapping DNA modifications. **A** Bisulfite sequencing begins with the treatment of genomic DNA (gDNA) with sodium bisulfite, a chemical that converts unmethylated cytosines (C) to uracils (U), while leaving methylated cytosines unchanged. PCR-amplified DNA fragments are then sequenced to determine the methylation status of cytosines across the genome. **B** Single-molecule real-time (SMRT) sequencing monitors the incorporation of fluorescently labeled nucleotides by a DNA polymerase in real time. Each nucleotide addition emits a fluorescent pulse as a signal. The DNA sequence is determined by the pattern of these signals, and interruptions in the pulse sequence indicate the presence of covalent modifications within the template DNA. **C** Nanopore sequencing utilizes the movement of a single DNA strand through a nanoscale pore (nanopore). As the DNA molecule passes through the pore, it causes disruptions in the electrical current, which are used to deduce the DNA sequence and detect DNA modifications such as 5mC, 4mC, and 6mA

Genome-wide methylation analysis typically employs methods such as digestion with methyl-sensitive restriction enzymes (MSREs) combined with next-generation sequencing (NGS), offering insights into methylation patterns at specific genomic loci [30]. However, this approach is constrained by specificities of available enzymes, often necessitating complementary techniques, including whole-genome bisulfite sequencing (WGBS) [31] and methylated DNA immunoprecipitation sequencing (MeDIP-seq) [32]. WGBS, in particular, differentiates between methylated and unmethylated cytosines but struggles to detect 6mA without additional adjustments [33–36] (Fig. 1A).

The advent of third-generation sequencing technologies, namely single-molecule real-time (SMRT) sequencing and nanopore sequencing, has revolutionized epigenome studies by directly detecting methylation patterns without chemical treatments. SMRT sequencing offers precise profiling of bacterial epigenomes through long reads and unique fluorescent signals during DNA synthesis (Fig. 1B), although it can be resource-intensive compared to nanopore sequencing, which reads DNA molecules via ionic current disruptions [37–41] (Fig. 1C). Notably, nanopore sequencing’s real-time detection capability is complemented by bioinformatic tools like Nanopolish and DeepSignal for accurate methylation pattern analysis [42, 43]. Additionally, tools like Nanodisco and MicrobeMod leverage comprehensive training datasets for precise methylation typing across different bacterial species [44, 45].

In comparing these technologies, PacBio SMRT sequencing stands out for its high accuracy and direct methylation detection capabilities, offering comprehensive insights into bacterial epigenomes, including precise identification of 6mA and 4mC modifications. However, its higher cost and lower throughput may limit its accessibility. On the other hand, Oxford Nanopore Technologies provides a more cost-effective and portable solution, ideal for large-scale studies and in-field research with higher accuracy probabilistic calls for a range of modifications, including 5mC, 4mC, and 6mA. The choice between these platforms depends on the project's specific needs, including budget, accuracy requirements, and the type of methylation being studied [46].

To further elucidate bacterial epigenomes, CRISPR-based technologies can introduce or modify epigenetic marks at specific genomic loci [47, 48], allowing researchers to explore the functional consequences of specific epigenetic modifications. Additionally, a bacterial epigenome database like REBASE provides curated information on DNA and RNA modifications in bacteria [49]. These methodologies and resources continue to evolve, contributing to our understanding of bacterial epigenomes and their roles in gene regulation, virulence, adaptation, and other critical biological processes in bacteria.

### Bacterial epigenetics in microbial systems and synthetic biology

The epigenetic status of bacterial genomes has been linked to various microbial phenotypes, such as antibiotic resistance and pathogenicity [18, 21, 50]. For example, DNA methylation has been demonstrated to influence the behavior of individual microbial species within microbial consortia involved in bioremediation [51]. Furthermore, several studies have illustrated the close association between the evolution of antibiotic resistance in bacteria and DNA methylation, along with alterations in gene expression. By employing an efflux pump regulatory network (EPRN) model in *E. coli* strains, Motta et al. demonstrated that the epigenetic inheritance of transcription rates in genes related to EPRN is essential for acquiring adaptive resistance within a bacterial population [52]. DNA methylation within promoters of virulence genes in *Mycobacterium tuberculosis* and *Campylobacter jejuni* has been identified, offering potential targets or biomarkers for drug discovery and clinical diagnosis [53, 54]. Additionally, DNA cytosine methylation can be employed to control physiological processes and antibiotics production in several bacterial strains. For instance, the initial study that reported associations between DNA cytosine methylation and differentiation in *Streptomyces coelicolor* highlighted the role of cytosine methylation in governing both morphological differentiation and actinorhodin production within this microorganism. This discovery revealed new layers of complexity in gene expression and regulation within *S. coelicolor* [55]. Additionally, in *Streptomyces roseosporus* L30, an industrial strain responsible for producing daptomycin, a 4mC MTase named SroLm3 exerts a comprehensive regulatory effect on secondary metabolism [56]. The activity of SroLm3 regulates secondary metabolism in *S. roseosporus*, thus influencing daptomycin biosynthesis. These studies have demonstrated that modification of DNA methylation patterns in *Streptomyces* can lead to a metabolic switch, contributing to the enhancement of secondary metabolite production in *Actinomyces*.

**Fig. 2:**
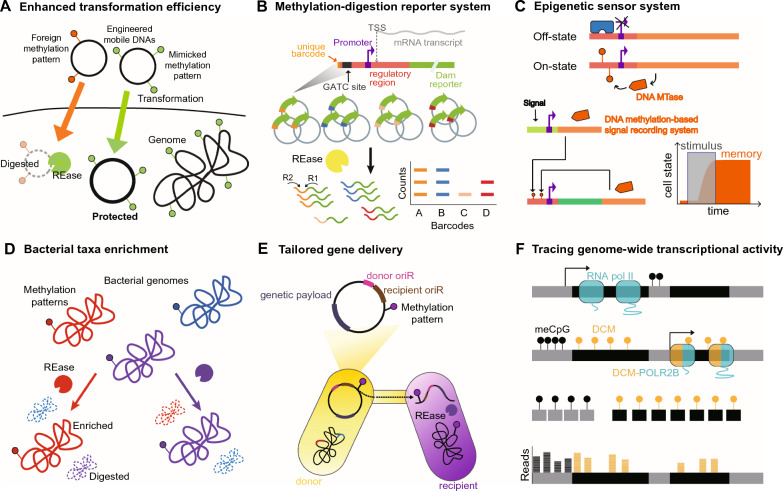
Potential applications of bacterial epigenetics in microbiome engineering. **A** Mimicking host DNA methylation patterns and recoding DNA sequences with synonymous nucleotide modifications or single-nucleotide polymorphisms (SNPs) have facilitated the evasion of R-M barrier in bacteria. **B** DNA MTases can serve as reporter systems for monitoring protein expression levels, achieved by their integration with methylation-sensitive restriction enzyme digestion and subsequent high-throughput sequencing. **C** Epigenetic regulatory systems utilizing DNA methyltransferases have been devised to sense targeted signals and maintain an epigenetic memory of cellular states. **D** Natural bacterial DNA methylation and methylation-sensitive Type II REases have been harnessed to selectively enrich genomes of specific bacterial subgroups within microbiomes. **E** Type IV secretion systems (T4SS), combined with DNA methylation patterns, could offer precision in gene transmission to specific microbes. The use of DNA methylation patterns ensures that gene delivery is confined to targeted microbial clusters, thereby enhancing target specificity. **F** A fusion protein of DCM (DNA cytosine methyltransferase) and the RNA polymerase subunit b, along with methylated DNA sequencing, has been employed to trace genome-wide gene transcription over time

The exploration of DNA methylation in bacterial genomes has highlighted the versatility and potential of epigenetic alterations for manipulating microbial behavior and biological processes across diverse applications (Fig. 2). One notable application has been the use of bacterial epigenetic modification to ensure the stable maintenance of plasmids within bacterial hosts. Despite extensive research efforts to efficiently manipulate non-model microbial strains, low transformation efficiency remains a significant challenge. This bottleneck mainly arises from the presence of bacterial restriction-modification (R-M) systems, which serve as defense mechanisms against foreign DNA [27]. Bacterial R-M systems employ specific enzymes that recognize and cleave foreign DNA lacking the host-specific methylation pattern. To distinguish between their own DNA and foreign DNA, DNA MTases within the R-M systems methylate bacterial self-DNA at specific recognition sites. This methylation prevents self-cleavage by their cognate restriction endonucleases (REases). Consequently, the bacterial restriction barrier leads to reduced transformation efficiency during genetic experiments, as only a portion of the introduced DNA can evade cleavage and be stably maintained in the host.

To enhance the chances of successful transformation, strategies involving the mimicking of host DNA methylation patterns have been proposed (Fig. 2A). After analyzing the methylome to identify functional R-M systems and DNA methylation sites in a target bacterium, DNA MTases can be isolated from the target bacterium or related species and then used to methylate a DNA template to be delivered [57]. This modified DNA can then be incorporated into the target bacterium without restriction, significantly increasing the transformation efficiencies. Additionally, multiple DNA MTases from difficult-to-transform target bacteria can be co-expressed in *E. coli* strains lacking known R-M systems [58]. This process enables shuttle vectors in the *E. coli* strains to adopt the methylation patterns of the target bacteria, allowing the plasmids derived from these hosts to evade R-M barriers of target strains and facilitating genetic manipulation. This approach described that mimicking DNA methylation patterns can overcome transformation challenges in bacteria that are difficult to transform, including *Bacillus amyloliquefaciens* and *Nitrobacter hamburgensis*. Since expressing multiple different DNA MTases for in vivo DNA methylation is quite challenging, Vento et al. recently reported the use of cell-free transcription–translation for recreating DNA methylation patterns in in vitro reactions to facilitate efficient multiplex DNA methylation and testing [59]. The advantages of mimicking DNA methylation patterns include increased transformation efficiencies and more stable maintenance of introduced DNA [60, 61]. Apart from mimicking DNA methylation patterns, another approach involves removing sites recognized by R-M systems from plasmid DNA to bypass R-M systems [62]. Following genome and methylome analysis with SMRT sequencing to define the bacterium’s R-M target motifs, these motifs are eliminated by recoding of the DNA sequence template via single-nucleotide polymorphisms (SNPs) or synonymous nucleotide modifications. This creates an R-M silent DNA template termed SyngenicDNA, which could enable microbial genetic system design not restrained by innate R-M systems. Hu et al. also identified potential REase recognition motifs by screening under-represented short DNA sequences, presenting a novel strategy to significantly enhance bacterial transformation efficiency [63]. These approaches address challenges posed by R-M systems, potentially transforming the landscape of microbial genetic engineering.

DNA methylation can also be employed to comprehensively characterize the transcriptional and translational activities of various regulatory sequences in bacteria. For instance, a versatile technique known as expression level monitoring by DNA methylation (ELM-seq) has been devised to uncover critical determinants in bacterial transcription and translation [64]. ELM-seq employs DNA adenine methylase from *E. coli* as a reporter system to monitor protein expression levels by combining with methylation-sensitive restriction enzyme digestion and high-throughput sequencing (Fig. 2B). Demonstrating its utility in the genome-reduced bacterium *Mycoplasma pneumoniae*, ELM-seq facilitated the in vivo identification of sequence elements that play essential roles in bacterial promoters and 5’-untranslated regions (UTRs). We anticipate that ELM-seq will be valuable for characterizing the regulation of diverse non-model bacterial species, especially for anaerobes where green fluorescent protein (GFP) and fluorescence-activated cell sorting (FACS) cannot be readily applied. Given that epigenetic modifications within bacterial genomes can impact gene expression levels, gaining a deeper understanding and control of these modifications holds significant potential for microbial engineering.

Various biological sensors and memory devices have been developed using bacterial epigenetic mechanisms. As an example, researchers constructed an epigenetic switch based on *opvAB* operon, which regulates bistable gene expression in *Salmonella enterica* through DNA adenine methylation [65]. Combining synthetic circuit design with epigenetic mechanisms, a synthetic epigenetic memory system capable of sensing transient stimuli and storing information as reversible DNA methylation patterns across many cell generations was devised in *E. coli* [66]. Maier et al. employed an engineered adenine-N6 methylation dependent DNA binding protein using the zinc finger chassis and the CcrM methyltransferase from *Caulobacter crescentus* to construct a bistable module for epigenetic memory systems (Fig. 2C). Based on the same architecture, Ullrich et al. further developed an epigenetic biosensor system with exceptional sensitivity to tetracycline [67], demonstrating potential utility of bacterial epigenetic modification systems for extensive signal storage and logical operations.

Bacterial epigenetics holds remarkable potential as an innovative toolbox for microbial systems and synthetic biology, yet navigating its complexities to achieve precise and predictable outcomes remains challenging. The alteration of epigenetic marks may result in unanticipated behaviors in engineered bacteria [68, 69]. This unpredictability, coupled with the current lack of standardization in epigenetic engineering techniques, poses significant challenges to achieving consistent results across different experimental conditions. Despite these hurdles, advancements in our understanding and continuous improvement of epigenetic engineering methods will significantly enhance our capabilities in precise characterization and manipulation of diverse microbial species.

### Emerging applications of bacterial epigenetics in microbiome research

Bacterial epigenetics, especially DNA methylation, offers a promising avenue for understanding and regulating microbial community dynamics to modulate microbiomes effectively. Leveraging bacterial DNA methylation patterns combined with SMRT sequencing, Wilbanks et al. have identified strain-specific DNA methylation patterns on metagenomic contigs, significantly enhancing the quality and comprehensiveness of metagenome-assembled genomes (MAGs) [70]. This capability arises from the distinct methylation patterns of various bacterial species, offering a more accurate representation of the microbial community present within the microbiome. Moreover, techniques like REMoDE (Restriction Endonuclease-based Modification-Dependent Enrichment) and mEnrich-seq, developed by Enam et al. and Cao et al., respectively, enable selective enrichment of specific bacterial taxa from metagenomic DNA before sequencing [71, 72]. These methods use natural bacterial DNA methylation and methylation-sensitive restriction enzymes to target and amplify genomes of interest (Fig. 2D), providing a cost-effective way to characterize microbiome members and improve microbiome sequencing coverage. Integrating these approaches deepens our understanding of microbiome dynamics.

Microbiome engineering often involves introducing specific genes into target bacterial species to alter their functions and physiological phenotypes. Horizontal gene transfer (HGT), crucial for the rapid development of new phenotypes like antibiotic resistance, could play a significant role. Shin et al. demonstrated HGT of DNA methylation patterns into bacterial chromosomes and its potential in cell phenotype programming and bacterial adaptation [73]. Type IV secretion systems (T4SSs) have been utilized for conjugative DNA transfer and genetic manipulation of diverse non-model microbes and microbial communities [10, 74]. While cell-surface displayed nanobodies were proven to be useful for rewiring target specificities of T4SS-based DNA transfer, challenges remain due to the limited availability of nanobodies for various bacterial species [75]. Additionally, the strategic manipulation of DNA sequences, including origin of replication, regulatory elements, selection markers, has been explored to enhance the genetic engineering efficiency of diverse microbial species within the microbiome [76–79]. Designing DNA templates with customized methylation patterns to match those of target species could further improve genetic modification precision. When introduced into the microbiome, such templates, carrying appropriate modifications, could be selectively taken up by compatible bacteria, enhancing microbiome engineering’s specificity and reducing off-target effects (Fig. 2E). Furthermore, bacterial epigenetics could offer a non-invasive method to modulate microbiome member phenotypes without introducing exogenous DNA, potentially fine-tuning traits like metabolic activities or stress responses by delivering DNA MTase proteins capable of modifying the methylation status of targeted bacterial species in the microbiome via Type VI Secretion Systems (T6SSs) [80].

Recent advancements, such as the DCM-TM (DCM-time machine), provide tools for monitoring gene expression and enhancer activity changes at the genome scale, potentially applicable in microbial hosts (Fig. 2F) [81]. DCM-TM employs a fusion protein of DCM (DNA cytosine methyltransferase) and RNA polymerase subunit b to label active genes and enhancers with DNA methylation patterns. This labeling facilitates the subsequent examination of gene activity at later stages of development or differentiation. They offer insights into dynamic shifts in cellular states during processes like development or disease progression, contributing to a deeper understanding of the gene regulatory networks underlying such transitions. These highlight new opportunities to transform microbiome research for a wide range of applications in biotechnology, medicine, and environmental science.

### Diversity of DNA methyltransferases in the human microbiome and their potential applications

Restriction-modification (R-M) systems comprise DNA MTases that modify specific DNA target sites and REases that cleave unmethylated or foreign methylated DNA, are fundamental in defending against foreign DNA like bacteriophages [82, 83]. Classical R-M systems (Type I–III) all rely on both MTase and REase domains, yet they vary in molecular structure, sequence recognition capabilities, operational mechanisms [26]. R-M systems encompass various configurations, including single enzymes containing restriction, modification and specificity subunits for Type I; separate MTase and REase for Type II; and complexes with multiple modification and restriction subunits for Type III. In contrast, Type IV R-M systems utilize only REases to target and cleave foreign methylated DNA [84]. Solitary or orphan DNA MTases, which lack associated REases, also play a role in vital cellular functions, such as DNA replication, repair, and gene expression [85, 86], highlighting their broad impact on microbial physiology [19, 22, 87]. Gene alteration in R-M systems can shift the gene expression patterns in bacteria like *Helicobacter pylori* [88]. Studies on orphan DNA MTases in bacterial species such as *Photorhabdus luminescens* and *Vibrio cholerae* have demonstrated their significant roles in bacterial viability, motility, and virulence [50, 89].

**Fig. 3:**
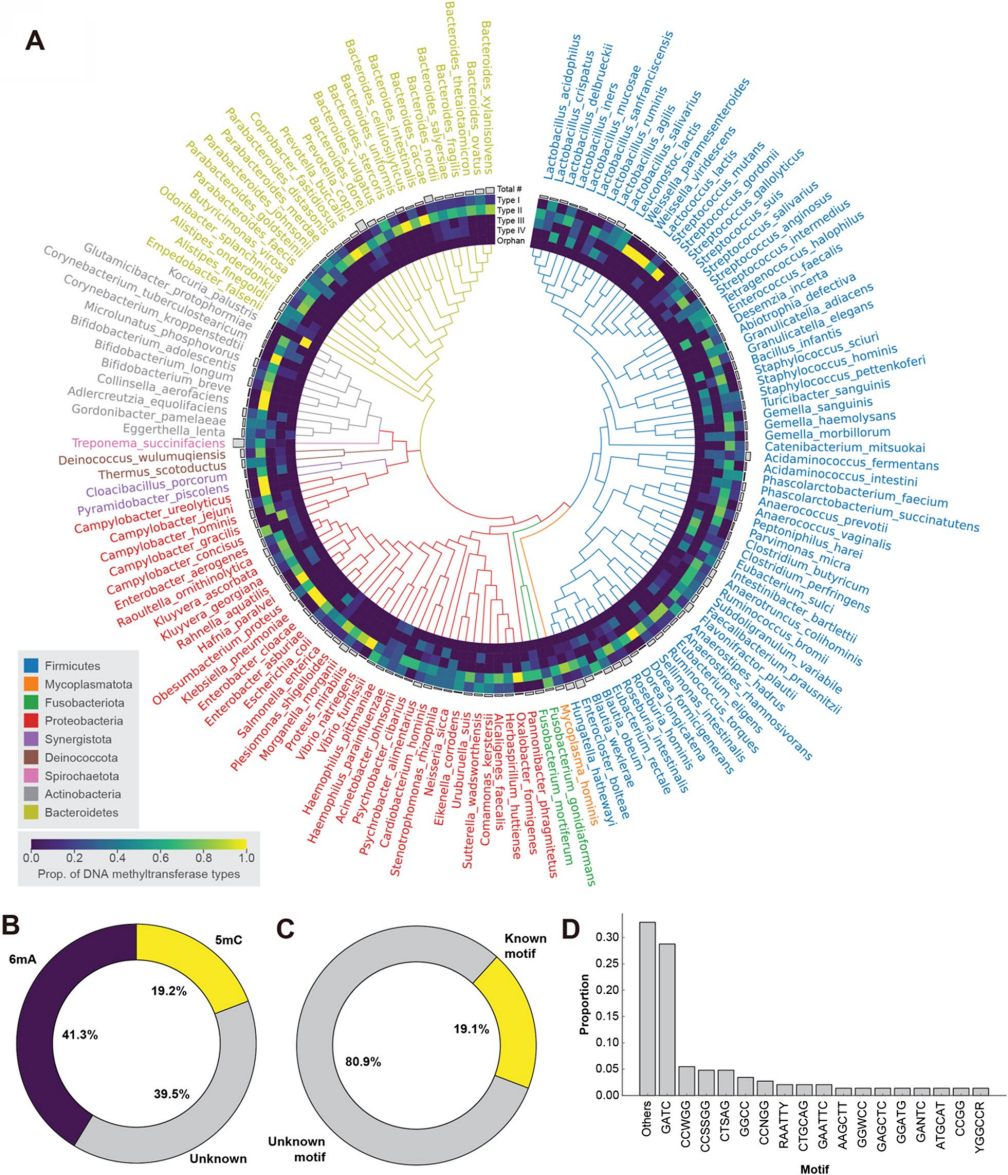
Diversity of DNA methyltransferases in the human microbiome. This figure classifies DNA methyltransferases from various bacterial species within the human microbiome using data from the REBASE database. A phylogenetic tree, color-coded by phylum, displays 150 bacterial species and is based on their 16S rRNA gene sequences. **A** A heat map details the distribution of Type I, II, III, IV, and orphan restriction-modification (R-M) systems across these species, while an adjacent bar plot shows the number of DNA methyltransferases per species, with *Treponema succinifaciens* registering the highest at 27. Donut charts (**B**) depict the prevalence of methylation types—5mC, 6mA, and unknown—across all methyltransferases and **C** the distribution of known versus unknown motifs within all R-M systems. **D** A bar plot enumerates the proportions of specific known methylation motifs for all R-M systems identified, with motifs appearing only once grouped as “Others”

The rich diversity of DNA MTases within the human microbiome provides opportunities for targeted community manipulation and deeper biological understanding. We surveyed the R-M system diversity across 150 bacterial species within the human microbiome, informed by NCBI and REBASE databases (Fig. 3A) [49]. The analysis indicates a predominance of Type II systems, followed by Type I, IV, and III, orphan DNA MTases. Additionally, we found that 5-methylcytosine (5mC) and N6-methyladenine (6mA) are prevalent methylation types, with a slight dominance of 6mA, while a significant fraction of methylation motifs remains unidentified (Fig. 3B&C). Among the identified motifs, GATC and CCWGG are the most prevalent, offering insights into the methylation landscape across the sampled species (Fig. 3D). We believe that the exploration of various types of MTases and methylation patterns, as well as uncovering their potential roles in shaping microbial phenotypes and community dynamics, will inspire innovative approaches to develop bacterial epigenetic modulators. These could have profound applications in microbiome engineering, paving the way for new strategies in this field.

## Conclusions

This review has examined the applications of DNA MTases in microbiome engineering, highlighting their integral roles in restriction-modification (R-M) systems and their potential for precision control of microbial behaviors. DNA methylation has emerged as a central player in the regulation of gene expression, shaping bacterial transcriptomes, and influencing a multitude of cellular processes. As microbiome engineering advances, the need for innovative techniques to effectively manipulate microbial communities has become increasingly clear. We have presented several methods for characterizing bacterial genomes and epigenomes that facilitate the alteration of epigenetic marks, enhancing the transformation efficiency and rendering challenging bacterial species more amenable to manipulation. These methods also enable comprehensive profiling of microbial communities, resulting in high-quality metagenome-assembled genomes (MAGs) and targeted enrichment of genomic data from microbiomes. The rich diversity of DNA MTases within the human microbiome opens up a new dimension for bacterial epigenome exploration. The integration of bacterial epigenetics into microbiome engineering holds great promise as an emerging frontier in understanding and manipulating microbial community interactions. While the application of bacterial epigenetics in microbiome engineering is still in its infancy, ongoing research and the creation of specialized tools are paramount. With the continuous growth of our understanding of bacterial epigenetics and the refinement of techniques, we anticipate that bacterial epigenetics will open up unprecedented opportunities for microbiome engineering and its vast array of applications.
